# Design of a bilingual (FR-UR) website on the sensitive topic of sexual and mental health with Urdu speakers in a Parisian suburb: a qualitative study

**DOI:** 10.1186/s12889-024-18479-w

**Published:** 2024-04-17

**Authors:** Sabah Jaroof, Johann Cailhol

**Affiliations:** 1 Registered Nurse and Master’s in Public Health at Laboratoire d’Educations et de Promotion de la Santé, Université Sorbonne Paris Nord, Bobigny, France; 2 Infectious diseases department, Groupe Hospitalo-Universitaire Paris Seine Saint Denis, Bobigny, France; 3 Laboratoire d’Educations et de Promotion de la Santé, Université Sorbonne Paris-Nord, Bobigny, France; 4 French Collaborative Institute on Migration, Aubervilliers, France

**Keywords:** Participative approach, Pakistan, E-Health, Taboo, Sexual health, Mental health, Website

## Abstract

**Background:**

This article is a continuation of the Musafir study published in 2020. Following the results of this study, we designed an educational website with Urdu-speaking volunteers, using a participatory approach. This type of approach aimed at bringing out situated knowledge around taboo/sensitive topics such as sexual and mental health, by considering the cultural, religious, economic, family, and social background of young Urdu-speaking men. This approach allowed us to build culturally-appropriate content matching the needs of targeted population. We report here the lessons learned from our approach.

**Methods:**

Urdu-speaking volunteers were recruited via outreach strategies, for participation in focus groups. Four focus group discussions were conducted on three distinct themes: 1/ Sexual Health Promotion, 2/Hepatitis and sexually transmitted infections, and 3/ Mental Health. The focus groups were recorded, with the written consent of the users. Thematic analysis was conducted after transcription of the focus-group discussion.

**Results:**

We succeeded in mobilizing 4 Pakistani users, aged between 19 and 30 years. The group dynamics was very rich and allowed us to highlight numerous social aspects related to the importance of the group belonging, the family, and others points of view on these topics. Many Urdu vocabulary had to be redefined and revealed the extent of the pre-existing taboo.

**Conclusions:**

Notwithstanding the extreme difficulty of mobilizing an invisible target population on a sensitive topic such as sexual and mental health, our experience highlights the need to consider the knowledge of the people concerned. The participative approach allowed us to fit the content of our medium to, for instance: the collectivist type of society of the target population; the level of literacy in their mother tongue; and to the embodiment of some taboo in their vocabulary. Although time and energy consuming, our approach seems relevant and could be replicated to other communities.

**Supplementary Information:**

The online version contains supplementary material available at 10.1186/s12889-024-18479-w.

## Background

### Where do we start from: the Musafir study

According to the World Health Organization, Pakistan has the second highest prevalence of hepatitis C (HCV), after Egypt [1]. Europe, including France, is witnessing a recent increase of immigrants from Pakistan, represented mainly by young single men, some of whom are affected by HCV and sexually transmitted infections (STIs). Various studies [2] have shown that most documented HCV transmission in Pakistan occurs via health-related procedures (re-use of contaminated syringes and therapeutic injections) and intra-venous drug use [3]. However, in the majority of cases, the source of transmission remains unknown [3]. Unprotected Men having Sex with Men (MSM) practices constitutes a risk factor for HCV while its heterosexual transmission is exceptional [4].

The Musafir study [4, 5], conducted at the University Hospital of Avicenne in Seine-Saint-Denis, France, aimed at understanding representations of hepatitis and STI amongst a sample of Pakistani patients. Thirteen Pakistani participants were selected from those followed-up for chronic hepatitis C, B, and/or HIV. The study combined semi-structured interviews, a focus group discussion (FGD), and ethnographic observations and the authors used a deductive and inductive analysis method [5].

Findings [5, 6] highlighted the socio-economic situation of recent Pakistani migrants in France. The young men often lived in multi-family housing with no privacy. They lived in precarious conditions with unstable, short-term jobs that were poorly paid, often with an illegal status. Having left their families in Pakistan and living in difficult conditions, in addition to not speaking French, their isolation had an impact on their mental health. Their level of knowledge about the transmission of STIs and hepatitis was low. At the same time, the study highlighted risky behaviors, particularly unprotected sexual practices including MSM practices. The taboo surrounding their sexual practices and orientation is illustrative of the family, cultural, and moral norms in their society of origin. The study findings allowed us to reflect on health promotion intervention strategies with the target population, including the creation of a digital tool to lift the taboo related to sexual health.

### Taboo of sexual education and interest in a digital tool

In Pakistan, sexual and reproductive health education is inadequate to almost non-existent in the environment of individuals, whether in training curricula or through the family [7]. Sexualitý is perceived as a taboo subject. On the one hand, it is forbidden to have sexual relations outside of marriage [7]. On the other hand, broaching this topic, according to the representations of many Pakistanis, would encourage youth to engage in deviant behavior [7]. The multilingual website Zanzu, produced by Germans for a migrant audience, describes a major obstacle related to these themes on sexuality, perceived as taboo in the society of origin [8]: “*Sexual taboos, traditional myths, and the cultural definition of sexuality as exclusively intrafamilial can contribute to dissuade people in need of information from seeking knowledge themselves*” (authors’ translation) [8, p. 2]. Yet, accessibilitý to information is paramount [9] for individuals to understand and adapt their behaviors with respect to their health status.

In the Musafir study [5], the respondents (patients living with the Human Immunodeficiency Virus or hepatitis C/B) [5] emphasized, among other things, the desire for a digital health promotion tool in Urdu and French, including items on sexual health and also mental health, which would make it possible to preserve anonymity [10]. Developing such a tool would allow for unobtrusive communication on sensitive subjects [10, 11].

According to Ferron C., E-health is an “*easy form of access to information* [for an audience] *with low health literacy*” (authors’ translation) [12]. Thus, using a smartphone, Urdu-speaking users would be able to search for information about their health on a bilingual website [12, 13]. The bilingual Urdu-French nature of the site would allow allophones to access information, including information from health professionals who may not be able to translate all the information in real time [8]. In addition, the French section would allow users to familiarize themselves with French terms.

### Genesis of participatory website development

A piloting committee, composed of a user representative, Urdu-speaking health professionals, and non-Urdu-speaking health professionals, was created, based on the decision to develop a bilingual Urdu-French website.

The architecture of the digital site was developed around the three specific themes that emerged from the analyses of the first Musafir phase [5]: sexual health, hepatitis and STIs, and mental health. Each theme was first worked on in a co-design workshop, in small groups composed of two health professionals (ethnic matching: one Urdu-speaking and one non-Urdu-speaking) and a concerned person (Additional file 1). The persons concerned were recruited within the Avicenne Hospital.

The workshops on the topic of sexual health focused on more general aspects at first. It was important not to be in conflict caused by not respecting everyone’s perceptions on the subject. Therefore, the topic of homosexuality was addressed but it was decided to not make it visible on the front page of the site.

Each of the three groups produced a detailed framework in terms of content to be developed for the website. In addition, since community support would be necessary in order for the digital tool to be received favorably, we decided to implement a completely participative approach, by which future users of the site could contribute their knowledge in the co-conception.

Furthermore, Ahung’s study on a digital tool noted a “*lack of research and discussion on successful strategies to create support for and overcome resistance to its implementation in schools and communities*” [7, p. 129].

**Fig. 1:**
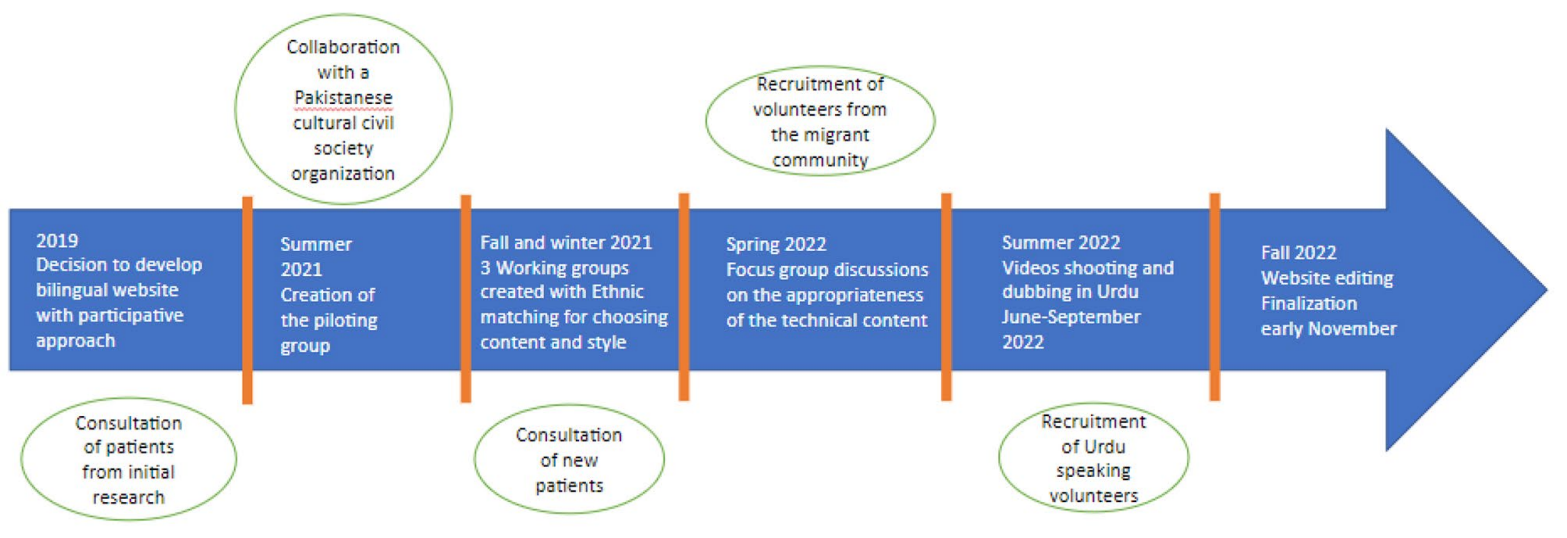
Website development timeline

The further step was constituted of FGDs with participants recruited from the Pakistani community living in Seine- Saint -Denis, in order to examine the content of the framework developed in previous workshops. The entire process is presented in the Fig. 1.

The objective of our paper is therefore to describe how knowledge emerged during the FGDs, as well as analyzing the levers and obstacles to this emergence, on the taboo subject of sexual and mental health within a community, when education on these topics is almost non-existent.

## Methods

### Study design and rationale

This is an action-research project that uses a participatory approach and qualitative data collection tools via focus groups. The participatory design consists of “*considering the design phase as a mutual learning process between patients, health professionals and designers/developers*” (authors’ translation) [14, p. 71]. This implies adopting a person-centered approach.

In an article on sex education in Pakistan, *“the authors also recommend that, when it comes to interventions involving controversial or taboo topics, the reasons for the low levels of community readiness should be investigated, and the interventions should be tailored with careful stakeholder involvement from the community so that the intervention is not dismissed outright*” [15, p. 8].

The participatory approach makes it possible to test the acceptance of such an action within the targeted community. Thus, the people concerned from the Pakistani community living in France shall agree with this project. Accordingly, the website is from the very beginning, incorporating the knowledge and representations of each stakeholder.

The participatory approach is a means, in our opinion, of achieving the appropriate exchange of knowledge: the experiential knowledge of the target audience versus the knowledge of experts.

*The group situation can facilitate a discussion on taboo subjects, due to the fact that the less inhibited participants draw the others into a dynamic that breaks the shynesś of the former. Joint participation can also provide mutual support in that it allows for the expression of feelings that deviate, possibly, from the cultural norm (or supposedly so in the researcher). This is particularly important in the case of research topics related to taboo or “stigmatizing” experiences (such as sexual harassment or violence).* (authors’ translation) [16, p. 241].

This refers to the idea, as conveyed by Ahung, that to confront conservatives and their denunciations of “*breaking the moral fabric of Pakistan*” [7, p. 134], a communication strategy must be developed, allowing for a dialogue on the taboo subject. “*Each form of resistance requires specific strategies and contextual knowledge*” [7, p. 135]. The focus group is a technique that aims to have people interact on a given topic related to the research question. The interaction can produce a richer collection; it is assumed that reality can be characterized by the interaction between people, instead of being collected by the perception of a single person, his or her emotion, etc. A FGD is therefore a method of investigation that aims to have people exchange ideas even if they do not agree. The facilitator tries to understand these disagreements, including the tensions between people. The intercultural dimension plays a key role in focus groups. Indeed, “*the way people express themselves refers to the idea that debates and arguments on a current topic are not an obvious social practice in our cultures*” (authors’ translation) [16, p. 241].

Taboo subjects can sometimes prevent focus group participants from interacting. However, this is not a limitation. As Kitzinger et al. point out, “*The group situation can facilitate discussion of taboo subjects, because the less inhibited participants draw the others into a dynamic that breaks down the shyness of the former. Joint participation can also provide mutual support, in that it allows the expression of feelings that may deviate from the cultural norm (or the researcher’s assumption that they do)*” [16, p. 241].

### Recruitment

Participants were recruited through several mobilization techniques. The inclusion criteria were being a young Urdu-speaking man who had recently arrived in France and being willing to participate in the FGDs.

To mobilize the young men, we implemented communication strategies such as the creation of posters and the distribution of flyers in places frequented by Urdu-speaking users (e.g., medical offices with Urdu-speaking doctors, restaurants, barbers in La Courneuve). We also launched an appeal on social networks such as Facebook and TikTok. We asked for the support of the French-Pakistani second generation in this recruitment phase. Finally, through the members of the project committee, an internal recruitment was also solicited at their professional workplaces (i.e., at the hospital, at information booth on access to care, and in shelters for young migrants). Also, one of the researchers (SJ) came from the Urdu-speaking community and held a position as an internal researcher. SJ used her “insider status” [17] to recruit participants directly and to communicate with them. SJ had linguistic, social and cultural resources that did not require the presence of a gatekeeper from the community [17].

### Data collection: focus group discussions

The FGDs were facilitated by a pair of non-Urdu-speaking health professionals (male or female, Muslim or non-Muslim, Caucasian or not) and an interpreter/translator of French origin, non-Muslim, who spoke Urdu as a second language. The health professionals were three infectious diseases specialists from the Avicenne Hospital who had already worked with this type of population. A non-participant observer, also Urdu-speaking, of first-generation Pakistani origin, was present at all FGDs.

The FGD guide was written based on the first Musafir study, and the technical content for the digital site was developed and selected in workshops by subgroups (see background and Additional File 4). Before the FGDs, a debate took place with the interpreter about the difficulty of translating certain words about sexual health into Urdu, despite the use of several translator websites. The difficulty was shared with the participants. We decided to describe certain illnesses using precise symptoms and used English vocabulary in order to eventually find the corresponding words in Urdu.

Throughout the FGDs, we worked with the participants on the following:

- their representations of the three themes,
- their life experiences,
- their shared understandings of the health behaviors adapted by themselves and their peers, and.
- the dissemination of content created for an evaluation.

The FGDs were recorded using a voice recorder with the agreement of the participants and then transcribed anonymously. After the transcription, the audio recordings were destroyed.

Field notes were taken during the FGD on behaviors and attitudes related to nonverbal communication and group dynamics, as well as the observer’s personal reflections.

The FGD were conducted in the Educations et Promotion de la Santé research unit in Bobigny. The premises are located at the university level, contributing to the confidence of the participants in the collaborative work process. Creating a space for discussion in a favorable location encourages the expression of everyone’s knowledge and experience.

Furthermore, in the collaborative approach, we decided to make a financial contribution to the participants for their work and time.

### Reflexivity and validity of the results

In order to give voice and space to the participants, the posture of the health professionals present in the FGD is crucial. A posture that leads to reflexivity on the part of the health professionals and that envisages that knowledge is to be constructed. In this phase, it is necessary to put aside the positivist posture of scientific knowledge and to make room for the knowledge of the people concerned, including their experiences. All the health professionals who contributed to the FGDs were fully aware of the need for this posture and were used to it by virtue of their duties.

This work benefited from a triangulation of data. We relied on data from the literature on the conduct of focus groups on culturally taboo subjects, on reflexive analyses from non-participant observations of the FGD, and on the cross-interpretation of the FGDs data by two people (JC and SJ), all of which considered gender, ethnicity, and social class domination relationships.

### Ethical framework

This study is part of the research protocol that received approval from the National Institute for Health and Medical Research (INSERM) ethics committee (IRB0000388, approval number 18–471, April 2018). All participants were informed in Urdu that they could choose not to answer any question and could leave the FGD whenever they wanted. Oral and written consent was obtained before each FGD for the recording.

Given the legal status of the participants, their socio-professional situation, and the sensitive topics discussed in the FGD, we took the time to explain the Musafir project, with the qualitative study conducted in 2018, the objectives of the stage in which the participants were engaged, and the purpose of this study. We also emphasized the non-judgmental nature of the research, i.e., that we were not there to point out their behaviors and that we wanted to discuss the content in a way that met the needs of the Urdu-speaking communities they represented. The telephone contact was made by one of the researchers (SJ), a native Urdu speaker.

### Method of analysis

An inductive approach, the grounded theory, was used for data analysis. We followed a classic step-by-step approach. We first familiarized with the transcripts by reading them several times. Then, SJ proceeded with a manual open-coding. Generated codes were validated by JC for one focus-group. SJ and JC followed with an axial coding, grouping codes into categories, further regrouped into 2 themes in the last step, the selective coding. SJ analyzed the Urdu part of the transcripts so as to not lose any meaning. JC was also present at the various FGDs and used the English part of the transcripts to validate SJ codes/categories and themes. Eventually, SJ and JC then reviewed the entire coding process against the transcripts and observation notes. Critical reflection was carried out throughout our analysis.

### Overcoming recruitment challenges

Implementing a participatory approach in this project was not easy, and mobilizing Urdu -speakers required the use of different means of communication, all culturally appropriate. For instance, during the flyer conception, we chose not to use the term “sexual health” or “mental health” so as not to block user participation (see Additional files 2 and 3). Furthermore, the major facilitating factor in this recruitment was that one of the researchers (SJ) was Urdu-speaking. SJ could directly manage interactions on the Facebook groups and be in contact with potential participants, hence helping in trust-building.

Eventually, four participants between eighteen and thirty years old were recruited. Their profiles are summarized described in Table 1. Names have been pseudonymized.

**Table 1:** Profil of the participants

	Geographical origin	Educational level in Pakistan	Level of French comprehension	Administrative status in France	Year of arrival in France	Recruitment source	Participation in focus groups
Basim	Urban Punjab	Master’s degree Native Urdu-speaker (advanced level)	Beginner level : Can understand but don’t speak fluently	Residence permit for work	2018	Second generation network	Focus group : 1, 2 and 3
Shafir	Rural Punjab	No degree Native Punjabi-speaker (Intermediate Level in Urdu)	Beginner Level	Resident permit for work	2018	Professional Colleague working with young migrants	Focus group: 1 and 2
Shams	Rural Punjab	Bachelor degree Native Urdu-speaker (Advanced level)	Intermediate level	Refugee status	2021	Professional colleague working in Hospital	Focus group: 1, 2, 3 and 4
Moses	Rural Punjab	No degree Native Urdu-speaker (Advanced Level)	Beginner	Undocumented status	2021	Facebook group	Focus group : 2,3 and 4

### Focus group dynamics

A total of four FGDs were conducted between March and May 2022. Each FGD lasted three hours. During the first FGD on health promotion, the participants learned what the FGD was and its purpose. They were very attentive and listened to each other, and their behavior and concentration showed that they were engaged with the discussion.

In this collective work, we chose to apply progressive learning, so the information discussed was classified according to three levels: what must be known, secondary information, and additional information that participants could access according to their needs.

However, in terms of the topics covered, one participant (Shafir) did not express himself much or sometimes supplemented the answers of his peers.

From the first FGD, the participants wanted to talk about diseases. This highlights the importance of the “*biomedical”* side of health. The notion of prevention is not understood [5]. The participants had more time to speak than the health professionals or the interpreter. Real debates took place on the understanding of the information and the acceptance of the subject by their peers.

According to the participants, one thing was certain: a given subject may well be taboo to discuss in the community, but when people are on their own, they may go to the website to look at the information given. When asked what they would feel if we talked about sexual practices, there was a silence in the room. One participant had to re-explain the question to his peers. Another said, *“I get it, but it feels weird to talk about it […] he’ll have said it once, but he’s not going to talk about it all the time.”* Another participant expressed the idea that he could eventually talk to his doctor about his sexual practices if it affected his health, but not with those around him, i.e., his peers: *“When I am afraid, and I am afraid of the disease [i.e., STIs], I will tell the doctor, but I will make sure not to tell the others, to hide it from them.*” Thus, the participants would hide an illness for fear of being judged by their peers, given the taboo nature of sexuality. Sex outside of marriage is forbidden under Islam. The subject of homosexuality was very quickly discussed and put aside.

## Results

### Essential to adapt to the cultural context: no prioritization of health

As described, mobilization was difficult and getting the target population to adopt specific health behaviors on the topics of sexual and mental health was another challenge.

The participants pointed out that a recurrent behavior of Urdu- speakers is not to consult a doctor as long as a health issue does not prevent them from living in their daily life, or interfere with their work. This behavior is not specific to Urdu-speaking newcomers but concerns the majority of economic migrants. The prioritization of needs is different. Many do not have access to care or are not aware of the risks involved if they do not seek care in time [18]. Indeed, the participants indicated that Pakistani people do not feel concerned about their health until they experience a problem. The concept of prevention is not known by the population [5]. Moses shares his views on the construction of messages: “*It comes down to the idea that you don’t have much time. You don’t focus on it. You think as long as you don’t have anything, why should you do it and you avoid it. But if you tell them all these things, there may be some information about them, about their practice, about the last few days. And then they’re going to say, ‘I did this, so I should do that.*’”.

This input allowed us to rewrite the scripts using the phrase “*if you had a relationship, etc.*” In this continuum, the participants also mentioned the importance of including an “injunction” in the phrasing, reinforcing a paternalistic approach. According to them, their peers will not go for testing if we do not tell them “You have to get tested.”

For instance, Shams said, *“If you say ‘It would be nice to go [get tested],’ they will say it is not important. But if you say*, ***that it is necessary***, *then they will go.”* Importantly, he used the phrase *zaroorat hai*, meaning “it is necessary.”

To create the website content, we decided to select pre-existing videos and adapt them into Urdu. One of the videos explained consent through the image of a tea cup.^2^ The video was not very explicit. The participants laughed when they watched it, and Basim said, *“I say that if we set an example with tea, some people will not understand.”*

Faced with this implicit content, the participants recommended making a video with the example of touching. We observed the same phenomenon with the diagrams and other videos that used the second degree (‘askip’ video from the website ons’exprime.fr). The participants also advised that it was preferable to combine the diagrams with explanatory audio. Thus, we could understand the importance of the choice of format, content, sentences, and words. For a better reception and comprehension of the information, the dynamics of cultural adaptation are essential.

### Linguistic terms

Cultural adaptation also requires taking into account the vocabulary used and the lexicon available in Urdu on the subjects covered. Talking about sexual health requires first of all knowing how to use the terms in Urdu in order to find the vocabulary understood by the majority of speakers. For example, we spent time at the beginning of the project trying to find the right translation of the term “sexual health” into Urdu, *jinsi sehat*, so that the target population would understand the information. This precision/specification of the vocabulary around the topics discussed became a prominent theme in the discussions.

Literary Urdu terms were not understood, and some participants were surprised that such words exist. For example, the word for gonorrhea (*Suzak*) exists but is not used in Pakistani society. Except for Moses, the participants had never heard the term.

Therefore, it was preferable to use the word followed by the symptoms of the disease. Other words generated similar reactions. For example, upon hearing the Urdu word *mabaal* for urethra, the participants asked, *“It’s in English, mabaal?”* and *“It’s in Urdu mabaal*?” Shams recommended, “S*o if you write it clearly ‘chote peshab ke nali’ [the pipe through which one urinates], because one does not understand ‘mabaal*.’” Thus, the participants helped us to understand that the name of the organ would be misunderstood, like the term *suzak*. However, the function of the urethra is explained, people would understand more easily.

Other terms are borrowed from English. Indeed, the Indian subcontinent including Pakistan (Independence in 1947) was colonized by England, and traces of this colonization remain in the lexicon used by the Pakistani population. There is no Urdu term for “condom,” and Urdu speakers use the English word “condom” (*Kndom*). This echoes an observation by one participant on the place of English in Pakistan: “A*t the medical level, people don’t know much, while at the basic level they do.”* Some words were integrated through appointments with health professionals in the country of origin, such as *“x-ray,” “test,” “check-up,” “allergy,” etc.*

When we focus on the lexicon of private parts, we can note the frequent use of euphemisms. To designate the intimate part of the man or woman, when we translate the term into French, it gives us “*the part of the man’s or woman’s shame*” (*aurat ki sharamgah, mard ki sharamgah*). The term “sperm” can be translated by saying “*aulaad paida karne ke jarasim*,” which means “*the bacteria of children*.” The taboo around these subjects gives rise to specific lexicons with similar connotations, and this highlights the value of the lexicon in our content production work. These initial results have led to the creation of an Urdu-French lexicon on sexual health, which is essential for the social integration of the migrants.

### Community / family ties and mental health

Regarding sexual health, we had seen previously that when a person in the community has an STI, they hide the condition from their peers. During the last two FGDs, which addressed mental health, we realized that the place of families and friends was essential in the lives of the participants. Johann Cailhol [5] mentioned this importance in his article. Indeed, upon arriving in France, *“they mixed largely with other Pakistanis from Punjab who were recently arrived, due to, inter alia: their common language (Punjabi), pre-existing networks which provided access to employment and shared accommodation, and their ethnic/regional commonalities (reflecting the transnational adaptation of regional hostilities and ethnic loyalties in Pakistan, in the French context*).” [5, p. 7].

For the participants, relationships with family, friends, and co-workers impact their mental health, and therefore they need to establish good peer relationships. In this same logic, a participant evoked the question of the taboo concerning mental health “*because it is something that is taboo. Even within families, when we make fun of someone else, we say that he is crazy. It has a very strong weight in society if someone goes to a psychologist, if someone sees another person coming out of the psychologist’s office. It’s a real stigma in fact. So yes, we wait until the last moment*.”

This taboo on the subject of sexual health seems to exist for mental health as well in a society characterized by “collectivism” [19], where one’s place in the group is important. The gaze of the other conditions or influences health behaviors, and a person may avoid seeing a psychologist or a sexologist if he or she feels rejected and humiliated by society.

During our FGDs, participants expressed the idea that they could, within their families, discuss their problems, especially with their mothers. Basim described, “*When I am stressed, I talk to my mother, my brothers. They guide me positively: ‘Do it like this. It will get better.’”*.

Shams said, “*About mental health, there is no secret. You can do it with the family.”* This shows that it is possible to talk with one’s parents about subjects that are considered taboo. However, mental health and sexual health do not necessarily have the same representations and places within Pakistani society. These quotes show the limits of the individualistic/collectivistic classification [19].

## Discussion

Our paper suggests the importance of understanding the society in which the participants developed over the course of their lives as well as the place given to the subjects of sexual and reproductive health in their society of origin in order to create adapted content. Producing educational content with accurate information, with vocabulary that is adapted to the literacy level of the target population, as well as culturally appropriate, is essential. Communication strategies that include sociolinguistics must then be considered during health promotion interventions. The paper also shed the light on the challenge of mobilizing the target audience to participate in the conception of educational tools on culturally sensitive subjects. Overcoming these challenges requires the reflexive posture and lot of time to build trust.

### Collectivist versus individualist society

The FGDs included participants with common cultural values, thus facilitating exchange. The group tended toward a common production of knowledge (Triandis 1995) [20]. This work led us to review our conceptions of health, prevention messages, and the specificities of the target population, within a society described as “Western” and “individualistic” [19]. The individualistic society calls for a focus on the individual and a preference for his or her personal interests and needs over those of the group. However, the study participants’ behaviors beliefs, and values echo certain characteristics of the collectivist society [19].

The collectivist society, according to G. Hofstede [19], is characterized by the fact that the group is more important and the perceived needs are collective. It calls for loyalty to the group, with a sharing of resources. The feeling of “shame” is a concept that Hofstede attributed to the collectivist society. It can be linked to the taboo on sexual health in that an individual’s STIs are known to their group. Shame affects the different registers of life, whether it is the beliefs and values of these young adults, their relationships, their intimacy, their subjectivity, their culture, their existence, or the society in which they live [21]. It therefore affects the existence of the person. In the case of Pakistani youth, sexual shame is linked to social shame (being stigmatized because of one’s sexual practices) [22, p. 75]. Collectivist families attach importance to the notion of “face” [19]. In the FGDs, at several points, the participants defined the place of their families as central to their lives. In collectivist cultures, children are taught to occupy space with their peers and to agree with them about their opinions [19]. This influences communication and leaves little room for self-talk; the collective interest prevails and requires “*self-effacement*” on the part of individuals [19]. This can therefore influence the health behaviors of individuals.

However, Eva Green [23], in her analysis of this dichotomy between collectivist and individualist society, points out that it seems crucial not to categorize target populations in terms of this differentiation. The approach demonstrates the ethnocentric view. Indeed, this difference has been elaborated by a researcher [19] with a Western situated view on the issue, producing a knowledge about another society that he does not know internally.

*“Collectivism” often has a pejorative meaning, at least in Western countries: it evokes conformity, submission to group pressure, and loss of individualitý. It is also negatively connoted because of the valorization of individualism (Beauvois 1984, 2003; Beauvois & Dubois, 1988; Dubois, 1994; Sampsorç, 1977, 1988; Sinha & Füpathi, 1994). These negative connotations influence interpretations and blur the boundaries between scientific research and ideological positions”* (authors’ translation) [23, p. 155].

We may unconsciously take the risk of leading people to adopt behaviors that are standardized by our vision of public health and thus devalue their knowledge. In our interventions, we think of the individual and ultimately omit their interactions. Addressing the person in his or her individuality and considering the influence of his or her group is paramount for health behavior change. The ability of Pakistani youth to interact with their environment depends on their experiences with the topics being addressed and the “*level of development of their relational skills*” (authors’ translation) [14]. We created an empowering space [24], i.e., FGDs in an academic setting, with the aim of fostering the emergence of the participants’ knowledge which they were not aware of. These times of exchange valuing their experiences constitute a means of increasing their capacity to act, both on an individual and community level [22] (empowerment). The participants expressed that they appreciated the “FGD” format and they learned new things. They were “happy” to be able to help the community with this participation, which also denotes collectivism.

We also told them that we learned a lot from them, with their knowledge and recommendations. Indeed, re-questioning our public health practices was one of the consequences of the productive exchanges with the participants. These different elements can thus push us to adopt a real posture of reflexivity throughout our research work.

Moreover, concerning the participants, thinking about how we could achieve behavioral changes was interesting. Finally, this rather dichotomous social psychology can interact with psychological models of health. Various research studies [25] have been conducted in the field of public health on the place of models such as the Health Belief Model and the Theory of Planned Behavior in a population group. Applying these psychological models to health promotion interventions in order to bring about a change in behavior may be an effective route.

Further, what may be interesting is to build on this asset of the target population that may come from their “collectivist society”: “the *image of the non-Western person may be valued for their sense of community and spirituality”* (authors’ translation) [23, p. 156]. The concept of community health comes into its own in this situation, and its implementation needs to be further explored and not remain a mere concept. It should be noted, however, that community health is often misperceived, particularly in France, partly because of an amalgam with communitarianism.

### Challenges around the participation of the different actors

#### Related to the themes of the website

We have seen that it was difficult to mobilize this population on these taboo subjects, and paradoxically the perception of social utility was perceived *afterwards*. One of the four participants did not want to come to the focus group on STIs. As Judith Dean et al. [26, p. 911] stated: *“Addressing issues such as sexual health from a cultural perspective with a small, highly-visible, ‘hard-to-reach’ population has in the past resulted in the research being deemed ‘too hard’ to countenance.”*^3^ The community’s view of the participation of a person can be a limiting factor for individuals. Indeed, during the FGDs, the participants pointed out the importance of not being seen by their peers in a medical office specialized in sexology or psychology. Even going to a free STI information, diagnosis, and screening center can be stigmatizing since it is marked as a sexual health center. Going to such places would categorize them as “crazy” or “sick,” and they could face exclusion them from their communities. “*Fear that participating in research focusing on a socially and culturally sensitive topic may further add to discrimination and negative stereotyping already experienced post-settlement and create further unwillingness”* [26, p. 913].^4^

To counter this discrimination, we decided not to use the term “sexual health” in the recruitment poster and to broaden our target population by using the characteristic of being Urdu-speaking. This was to avoid stigmatizing a specific community for risky health behaviors. Elam reported, “*Research questions related to participants’ sexual health involve the exploration of personal and sensitive issues, and this can be particularly challenging with communities who identify this topic as culturally taboo and/or sensitive (Elam and Fenton 2003)”* [26, pp. 913–914]. Therefore, the participation of people in this intervention research seemed to us to be essential. Their presence was a resource because they are the people who are best able to provide us with leads for addressing sensitive issues in the community. The Musafir study allowed us to choose an adapted health promotion format to address the subject in a strategic way. It also allowed us to identify the obstacles related to this research (or implementation of the intervention) within a minority community. Establishing a climate of trust to implement our intervention is key. In this context, we did not insist neither on the theme of homosexuality.

### Related to the age / single status

Shafir’s age may have been one of the elements that hindered his ability to express himself. As Johann Cailhol [5] mentioned in his article, some people do not feel concerned by the problems targeted or the topics discussed around sexual health. Indeed, when we talked about reproduction, we asked participants if it was necessary to include content on the issue. The participants answered, *“Those who are married and want children, it’s important for them to know”* and *“It talks about having a baby, that I think they [Pakistani women] don’t know [how to procreate].”* These quotes highlight the fact that the men did not think they needed to know about such things because they were single.

### Taboo language: influence on vocabulary

In our discussions with the participants about content design, we focused on terms around sexual health. As Azhar Pervaiz et al. [27, p. 114] write, language is the reflection of the society in which we live: *“Language is the reflection of its corresponding culture and society. Where there is positive language like that of appreciation and respect, there are also its negative forms including racist and sexist language and the linguistics taboos.”* The child, in his first interaction with his parents, learns what the taboo is, the words not to say, the possible censures. This space expands as the child enters the social world and interacts with peers. The media and the various information channels will reinforce the taboo around certain subjects. These taboos become part of social norms and are thus internalized by people. As Pervaiz et al. says,

*In Oxford Advanced Learner’s English Chinese Dictionary (2004), taboo refers to cultural or religious custom that curbs people to do, practice, or talk about a particular thing. Taboo words are considered offensive and shocking by many people; i.e., they involve body, people’s race, or sex*.” [27, p. 114].

In the Christian context referring to “shame” and “guilt,” the designation of “shameful parts” is found in ancient literature and in medical discourse in the sixteenth century [28]. The purpose of this designation was to guarantee modesty. For instance, during dissections of bodies, the parts that were described as “shameful” were covered with a cloth. During this period, naming the parts was a source of hesitation, of reflection by the medical world in order not to offend morals or to promote eroticism [26]. Today in Western society, this designation no longer exists as science has made it possible to distinguish morality from knowledge [28].

Majidah Khanam et al.’s (2011) study on the knowledge and attitudes of medical graduates in Karachi on sexual and reproductive health reveals a resource-poor curriculum on this health specialty and a low level of knowledge among physicians on the topic [29]. This gap affects the management and care of individual sexual and reproductive health issues in the Pakistani health system. These data highlight the fact that the taboo, governed by norms, has an impact on health training curricula. Even health professionals, who carry prevention messages, receive little information about sexual and reproductive health, according to Khanam et al.’s study. We may wonder about the ability of second-generation Urdu-speaking doctors to discuss these topics with their patients of the same origin. As for the participants, during the focus groups, they thought that it was easier to discuss sensitive subjects with non-Urdu-speaking doctors. They also mentioned the lack of information on taboo subjects, prohibited by religion:

- Shams: “*It’s a concept. Religion tells us we shouldn’t eat pork, shouldn’t have sex before marriage, shouldn’t drink alcohol, but no one told us why we shouldn’t. We don’t have information.*”
- Moses: “*Yes, that’s what I was saying. It’s when you grow up that you learn, that you know why, but as a child, you’re away from these things, and in your mind, it’s ingrained that these things are bad, you mustn’t do them.*”

In 2011, a Pakistani doctor named Akhtar M. attempted to publish a textbook on sex education. After receiving death threats, he had to change the title of his book to “Special Problems for Young People” in Urdu instead of “Sex Education for Muslims” [30].

In today’s society, the context in which a term is used helps define whether it is “taboo” or not. In addition, in a society where the group is central, terms are influenced by the group effect. In order not to be rejected by one’s peers, the will to conform appears to preserve one’s honor (see [16] in reference to the collectivist society). As Pervaiz and al. quoted Trudgill in their article (1986, p. 28), “*The normal use of an item in a language is inhibited due to particular social values and beliefs”* ( (27, p. 115/116). Our work has shown the difficulty of naming certain body parts by the participants and the interpreter, as well as certain STIs, because the terms were not established by the participants and had associations with moral character. The terms are only used once a person develops a related pathology, according to the participants.

This result illustrates the extent of the taboo in a society in which certain information about sexuality (genitals) is not known by individuals. In Pakistan, Urdu is the official language. However, the country is multilingual due to its different regions (Sindh, Punjab, Balochistan, and the North West Frontier Province) [31]. The target population is predominantly from the Punjab region, according to the first Musafir study, and thus speaks Punjabi [5, 31]. Working with people from another culture and country predisposes one to have knowledge of their country of origin, beliefs, and values around these sensitive topics. Contextualizing words is a key approach in our content creation process.

The use of euphemisms to describe certain parts of the body reinforces the taboo nature of the subjects and the words used. According to Pervaiz et al., *“Euphemism involves the use of linguistic and paralinguistic features to avoid profane words, violence, offense, and embarrassment and to maintain honor”* [27, p. 117]. The question of honor subsists through taboo. We find this idea in collectivist societies [16] and Western societies in the 16th century.

In the literature, we can see from Malavige Lasantha et al. [32] that in the construction of measurement tools (i.e. questionnaires) on sexuality, researchers had to do considerable work to choose appropriate terms in five languages of the South Asian community (Hindi, Urdu, Punjabi, Tamil and Singhalese). *“The proper words used by the translators were found to be too difficult to be understood by an average man*” [32, p. 5]. Linguistic validation by the people involved allowed the production of a culturally understandable and acceptable scale. Without this, the information would not have been understood because the terms were not known. The scales were adapted to facilitate understanding. “*Many terms available were either too colloquial or too sophisticated, making them difficult to be understood by a broader group of men”* [32, p. 4]. That is, the terms were either too colloquial or too complex, and it was necessary to work with native translators. This aspect, which emerged during the course of our project, must be considered in the design of questionnaires and educational tools translated into the languages of origin of users of the health system in order to accurately convey information on sensitive subjects.

The difficulties we perceived in our work in translating words are similar to those described by Malavige Lasantha et al. [32, p. 5]: “*These difficulties were compounded in the present study as most of the terms describing sexuality-related issues in South Asian languages were either colloquial or slang*.” The taboo surrounding the subject accentuated the difficulty of translations. Thus, the importance of cross-cultural translation was highlighted by this study.

*In earlier studies of the taboo language, its use has been closely linked with the literacy/illiteracy of the speakers. The uneducated class does not have the access to rich vocabulary; so they are left to use the taboo words. But the researchers criticize it as being too traditional, claiming that the taboo language itself has diversity and involves innovation in the lexicon and its choices. Further, it has its own morphological and syntactical systems, especially when it comes to the swear words.* [27, p. 117]

In our case, we noticed that social status is not a determining factor in the knowledge of the terminology dedicated to sexuality. This work was possible because we had participants with different and ultimately high levels of Urdu literacy. We found that despite the high level of Urdu, the participants did not know a number of words related to sexual and reproductive health. This demonstrates the importance of the taboo around this topic. It is difficult to talk about a subject without naming it. The article on Zanzu discusses,

*the study ‘frauen leben 2 - Familienplanung und Migration im Lebens-lauf’ shows, for example, that due to the taboo surrounding sexuality—especially in connection with a low level of education—terms and designations of intimate body parts, physical processes, and sexual acts are missing.* (authors’ translation) [8, p. 2].

Further study is needed to confirm this initial link between the sexual health taboo and the sociolinguistics we relate.

## Limits

Our study has some limitations. The lexicon proved to be of primary importance in the discussion of the sensitive topics. The importance of having an interpreter whose mother tongue is the language of the website users seems to be necessary in a project of this type. Also, there is a potential loss of meaning through double translation (from Urdu to French and later to English). The topic of sexual health might have been more covered than mental health, due to group dynamics. We acknowledge need for deepening the mental health topic in further work, but due to the natural combination of both topics, we left them undistinguished here.

Thanks to this work with the four participants, we produced rich data. Nevertheless, it is important to mention that the number of participants was a limitation in this study.

Moreover, the Covid crisis changed our planning, which affected the level of commitment of the piloting committee members over the course of the project. Some people dropped out of the project. The commitment of all project members shall be evaluated over time. The small number of participants recruited for the FGDs highlights the difficulty of mobilizing the target public on this subject. We worked with volunteers who worked full-time and were still willing to dedicate time to this project. The interests of the actors/people involved/volunteers diverge and are not explicit in the end, which has an impact on the level of commitment of each. A final evaluation through semi-structured interviews could shed light on the perceptions of each stakeholder in this project.

## Conclusion

Working on a sensitive issue within a specific population requires specific strategies. For this, collaboration with the people concerned is a real asset in designing projects that are useful, acceptable and reflect *“the voice and social-cultural reality of the target community”* [26, p. 919]. Challenges emerge during the different phases, and the commitment of the people must be determined beforehand and maintained over the long term. This type of project requires time and organization, knowing that people in vulnerable situations do not perceive health as a priority need; hence, the importance of designing projects on culturally sensitive models [33] (Resnicow et al., 1999).

Cultural adaptation of tools and linguistic validation, as demonstrated in this study, can be factors influencing the project acceptability. Also, the presence of an Urdu-speaking researcher in the team allowed us to overcome cultural barriers without the use of a gatekeeper [17]. Through this collaborative work with participants, this study produced situated knowledge specific to their experiences. As Olivia Gross states, “*the development of situated knowledge is conducive to alternative solutions*” (authors’ translation) [34, p. 107] and therefore leads to the impact of practices. Creating the conditions for the emergence of participants’ knowledge, through techniques such as the FGD, makes it possible to establish a climate of trust between health care users and health professionals. This demonstrates the value of collaborative work and contributes to understanding the target population. Therefore, public health research conducted with populations with a migratory background shall take into account their first language and the surrounding context. The stabilization of a lexicon seems necessary in the acquisition of information and knowledge. This work on sociolinguistics around sexual and reproductive health (sensitive subjects) in Urdu, and in other foreign languages, whose culture is very different from that of France, constitutes research topics to be developed.

To continue this dynamic, the digital site will have a “forum” space in which users can exchange anonymously, without being judged. The forum will also be a place for the production of situated knowledge, which will be taken into account in the continuation of this project and in the development of specific interventions. This forum will make it possible to mobilize a larger number of users. Creating spaces for discussion, whether through focus groups, popular theater, or an online forum, contributes to the emergence of alternative solutions by people for their needs. These spaces are places where different disciplines (sociology, sociolinguistics, anthropology, ethnology, psychology, and public health) can intersect through a systemic approach. Developing “South Asian Studies” or “Black Studies” or “Disability Studies” by the people concerned in France presents a real challenge in understanding and accompanying these individuals with regard to their health behaviors, socialization, and history. Conducting this research as a concerned person with the same origins allowed SJ to have a legitimacy and facilities in particular in the recruitment of people, in the understanding of the studied problems, and in the planning and the organization of the various stages.

Being reflexive throughout our interaction with others in projects of this type is essential. The conditions under which the research is conducted will influence the results. It is therefore necessary to adapt to the needs of the populations and to think collaboratively about the strategies to be implemented in order to promote health behavior change.

### Electronic supplementary material

Below is the link to the electronic supplementary material.

Supplementary Material 1

Supplementary Material 2

Supplementary Material 3

Supplementary Material 4

## Data Availability

Authors are not able to share their raw data since participants disclosed very sensitive information. Participants were told that the transcripts will be read by SJ and JC only, and this prompted their trust.

## Footnotes

2 Video on consent with the example of a cup of tea: watch.

3 Dean et al. cite Hynes (2003), Ogilvie, Burgess-Pinto, and Caufield (2008), Smith and Pitts (2007), Wilson and Neville (2009).

4 Dean et al. Cite Colic-Peisker (2009), Ogilvie, Burgess-Pinto, and Caufield (2008), and Wilson and Neville (2009).

## Abbreviations

FGD: Focus group discussion
HCV: Hepatitis C Virus
HIV: Human Immunodeficiency Virus
MSM: Men having Sex with Men
STI: Sexually Transmitted Infections
