# Supplementary figures and images for: Design of a bilingual (FR-UR) website on the sensitive topic of sexual and mental health with Urdu speakers in a Parisian suburb: a qualitative study
Source: BMC Public Health. 2024 Apr 17;24:1075. doi: 10.1186/s12889-024-18479-w (PMC11025278; doi:10.1186/s12889-024-18479-w)

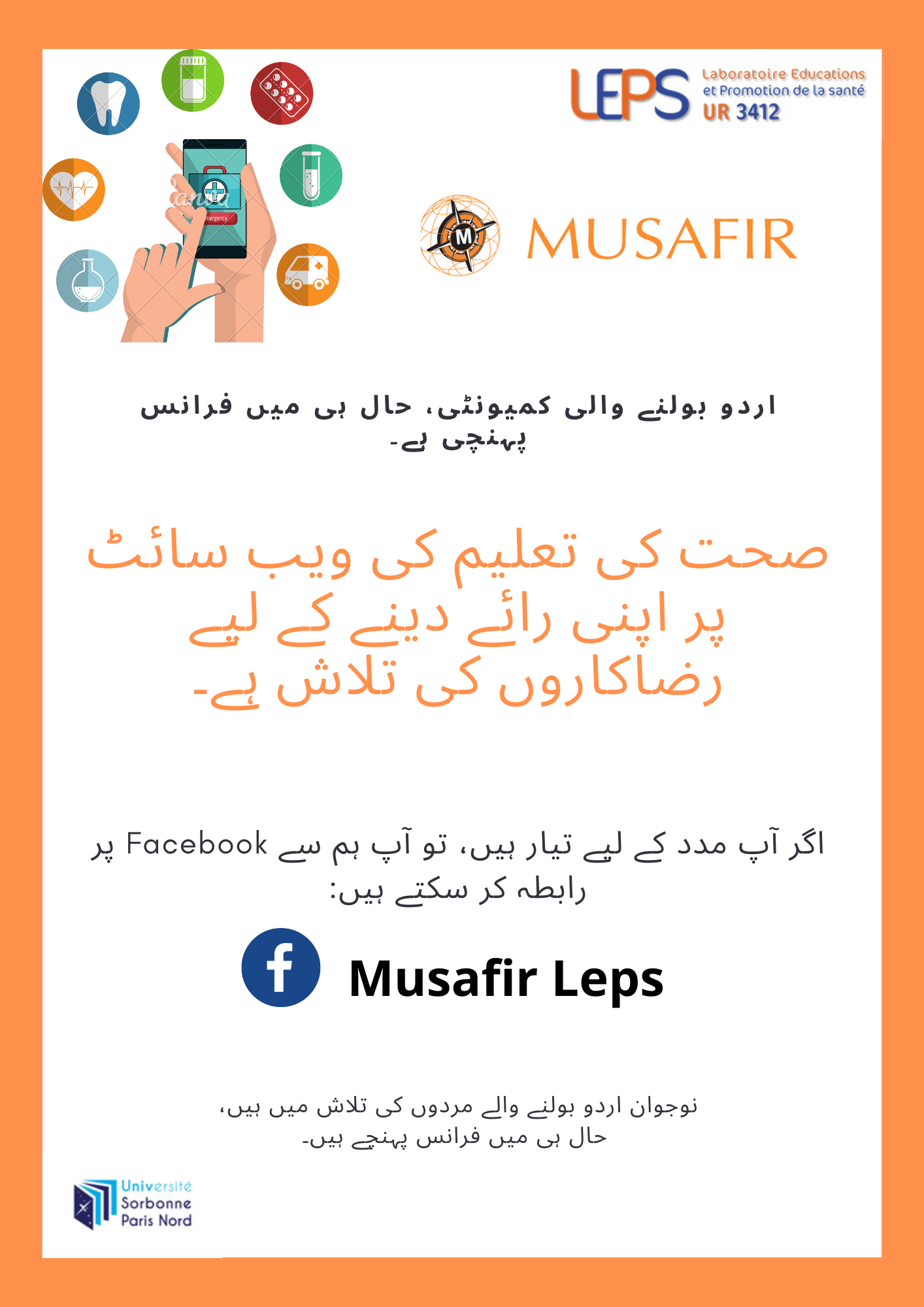

Supplement: Supplementary file 1 — Supplementary Material 1 [file 12889_2024_18479_MOESM1_ESM.png]

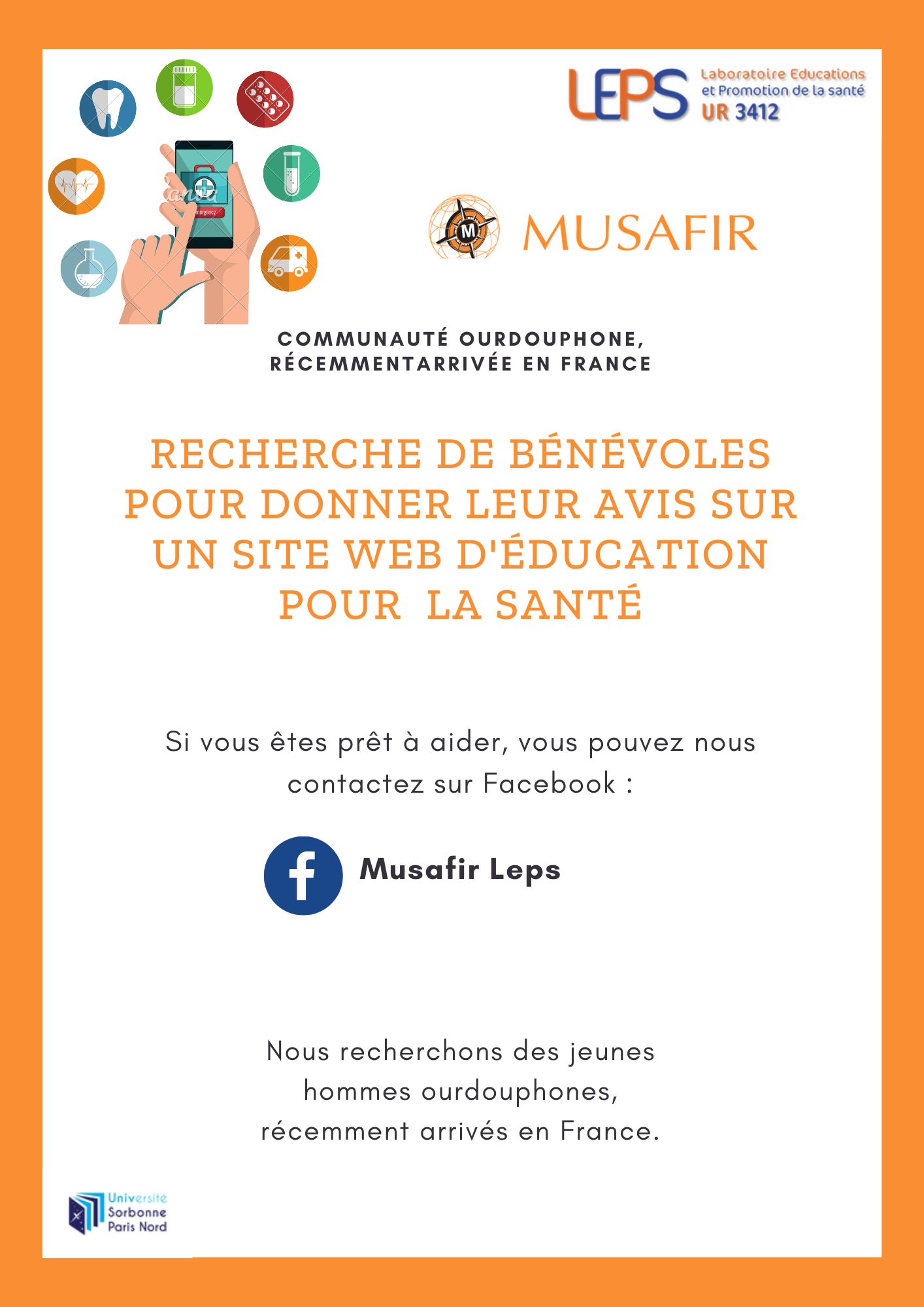

Supplement: Supplementary file 2 — Supplementary Material 2 [file 12889_2024_18479_MOESM2_ESM.png]
