# Supplementary material for: Design of a bilingual (FR-UR) website on the sensitive topic of sexual and mental health with Urdu speakers in a Parisian suburb: a qualitative study
Source: BMC Public Health. 2024 Apr 17;24:1075. doi: 10.1186/s12889-024-18479-w (PMC11025278; doi:10.1186/s12889-024-18479-w)
Supplement: Supplementary file 3 — Supplementary Material 3 [file 12889_2024_18479_MOESM3_ESM.docx]

**Methodology of the working groups**

To talk about the methodology, I based myself on the following article, entitled "The patient as an actor in e-health design: participatory design of a mobile application for cardiac patients", written by Gros-Jean, Bonneville and Redpath.

This work will focus on "an approach known as participatory design, which aims to produce an in-depth understanding of future users by involving them in the early stages of the technology design and development process (Simonsen and Robertson, 2013)". (from the article)

To involve the user, we have already used qualitative methods:

- Stage 1:

Publication of articles by Johann Cailhol & Nichola Khan, entitled "Chronic hepatitis and HIV risks amongst Pakistani migrant men in a French suburb and insights into health promotion interventions: the ANRS Musafir qualitative study" & "Are migration routes disease transmission routes? Understanding Hepatitis and HIV transmission amongst undocumented Pakistani migrants and asylum seekers in a Parisian suburb".

Semi-structured interviews (at MDM's CASO): the aim was to understand users' needs, their questions and their experiences of healthcare.

- Stage 2: Following these interviews, an analysis was carried out to better prepare the work to be developed.

We now need to carry out the other two phases:

- Stage 3: Organisation of participatory design workshops.
- Stage 4: Prototype produced and subsequently discussed between healthcare professionals and users for readjustment.

Characterise the features that we think the site could benefit from, so that users can make it their own.


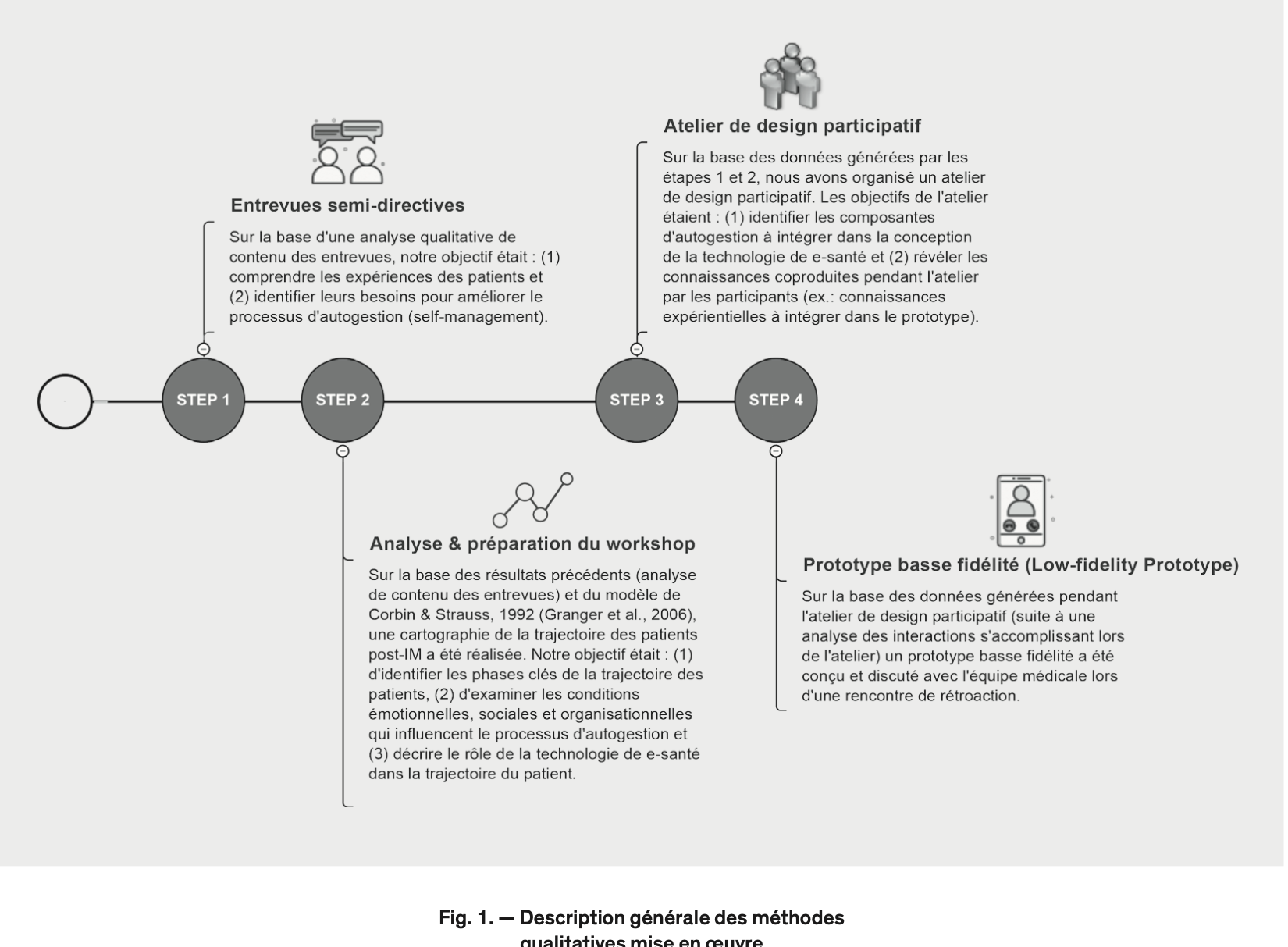


This design phase is a time for exchanging knowledge between the user and us, the healthcare professionals. It is a learning moment for both parties.

| **Objectives** | - Determining the materials and information to be placed on the site - Encourage user involvement during the workshop (the user must have a place like the rest of the team) |
| --- | --- |
| **Techniques** | Brainstorming, mind maps, Post-it games, etc. to be agreed with the team |
| **Purpose** | Create an initial prototype with the user using the media chosen for the website |
| **Participants** | Actors, Health Care Professionals |
| **Number of sessions** | 3 or more for each working group  Session 1: Without the user, with sorting of available resources  Session 2: With the user present, with a health literacy assessment, and drafting of the content outline  Subsequent sessions: with or without the user to produce the content,  Final session: with the user, to validate productions using the appended tool "Tool 4". |
| **Address for the sessions** | Sessions can be held at the Avicennes hospital or the Bobigny Paris 13 faculty. |

- When developing the content, it is necessary to start with a general approach, i.e. by saying "*We realized that the Pakistani population encounters a number of difficulties when they arrive in France concerning their health and their rights. In France, we do have rights, structures to help you take care of yourself and your health, etc.*".
- Then, from the general to the specific, focusing on the individual, for example: "*How can I get tested if I think I've put myself at risk during a sexual encounter? “*
- Participatory design workshops can be held, depending on the team's progress.
- Report back after each session.
- Informal evaluation by the facilitator, to be recorded in the minutes (see appendix).
- (see appendix) (assessment of patient satisfaction using a scale from 0 to 10 and self-assessment).

Comments :

- It's important to start from the fact that the users are going to be the real users, so we need to start from their daily lives: use narration=> life experience stories => actors in their own lives.
- A 3-minute* video clip will be created, focusing on the essential themes (early December),
- Resources must be sorted according to literacy level,
- Compensation for users
- It is important to take the time to find pre-existing content adapted to the target audience that will be translated => the aim is not to recreate new content.

*Content to be produced VIDEO CAPSULE :

The video clips will be three minutes long. A subject will be covered, for example, STIs:

First, we define what they are in a lexicon that users can understand;

Then the reasons why people contract these infections, i.e. WHY?

Then the solutions for preventing the onset of STIs, i.e. HOW.

For the video => this will be explained directly to the cameraman, with the support of texts, commented drawings and/or audio messages.

**Subjects :**

- **Health promotion**

Sub-themes :

- Starting with a general introduction to health, different types of health (mental, physical, sexual)
- Health literacy, Who can provide me with reliable information on sexual health (Health professionals? Peers?) (DEFINE THE PARTICIPANTS)
- Sexual health education including anatomy, STI prevention (Screening: Define first, then where to go for it) and rights (administrative and sexual in France => video done by Académie Populaire de santé)
- **STI**

Sub-themes :

Contextualised

- Viral hepatitis: Hepatitis B and C
- HIV
- Other STIs
- **Mental health**

Sub-themes :

Psycho-trauma :

- Acculturation
- Defining and managing emotions
- Identifying who you are as a person
- Our relationship with others,
- Migration, exile, trauma

Addictions : talking briefly about tobacco and drugs (no details)

**
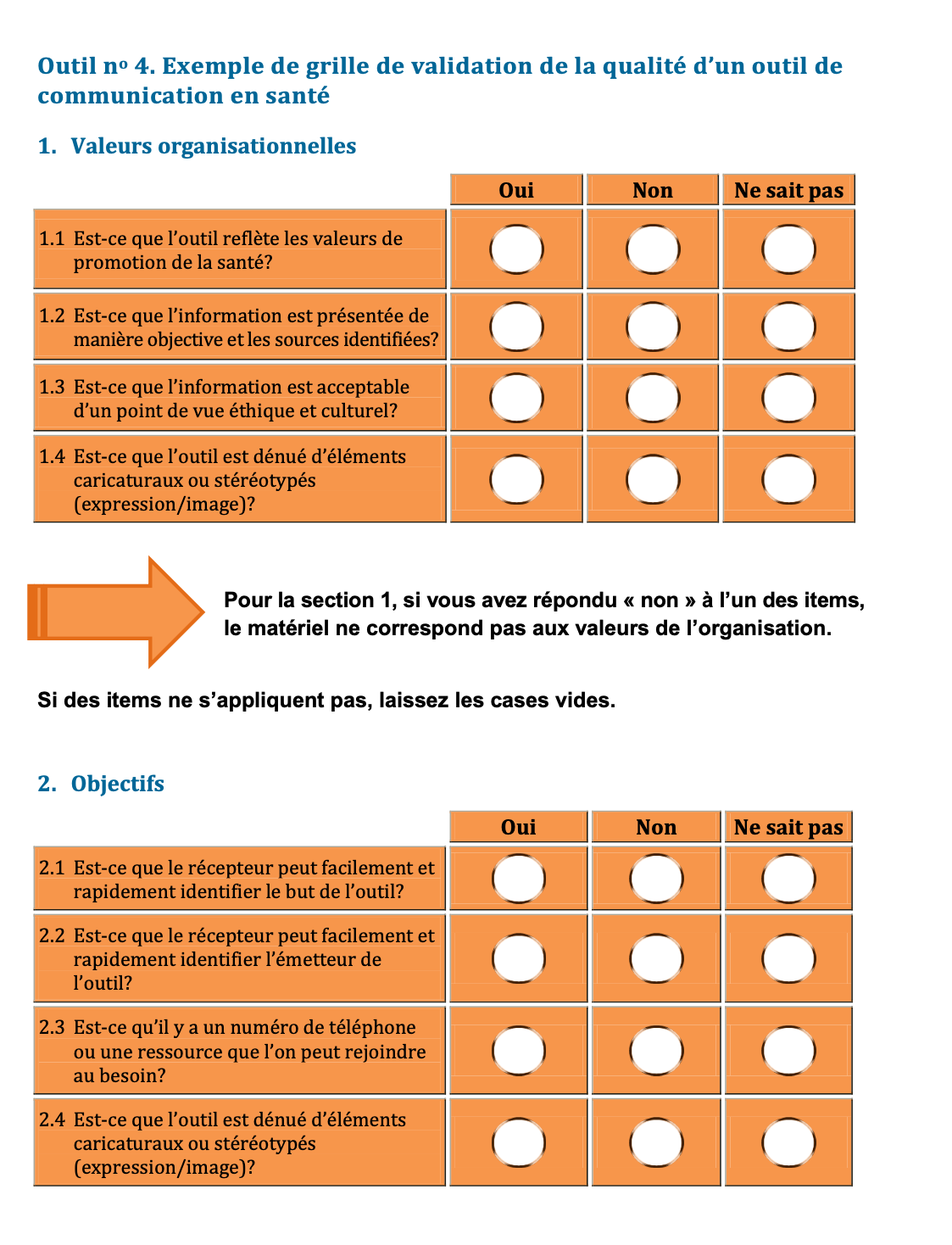
**



**
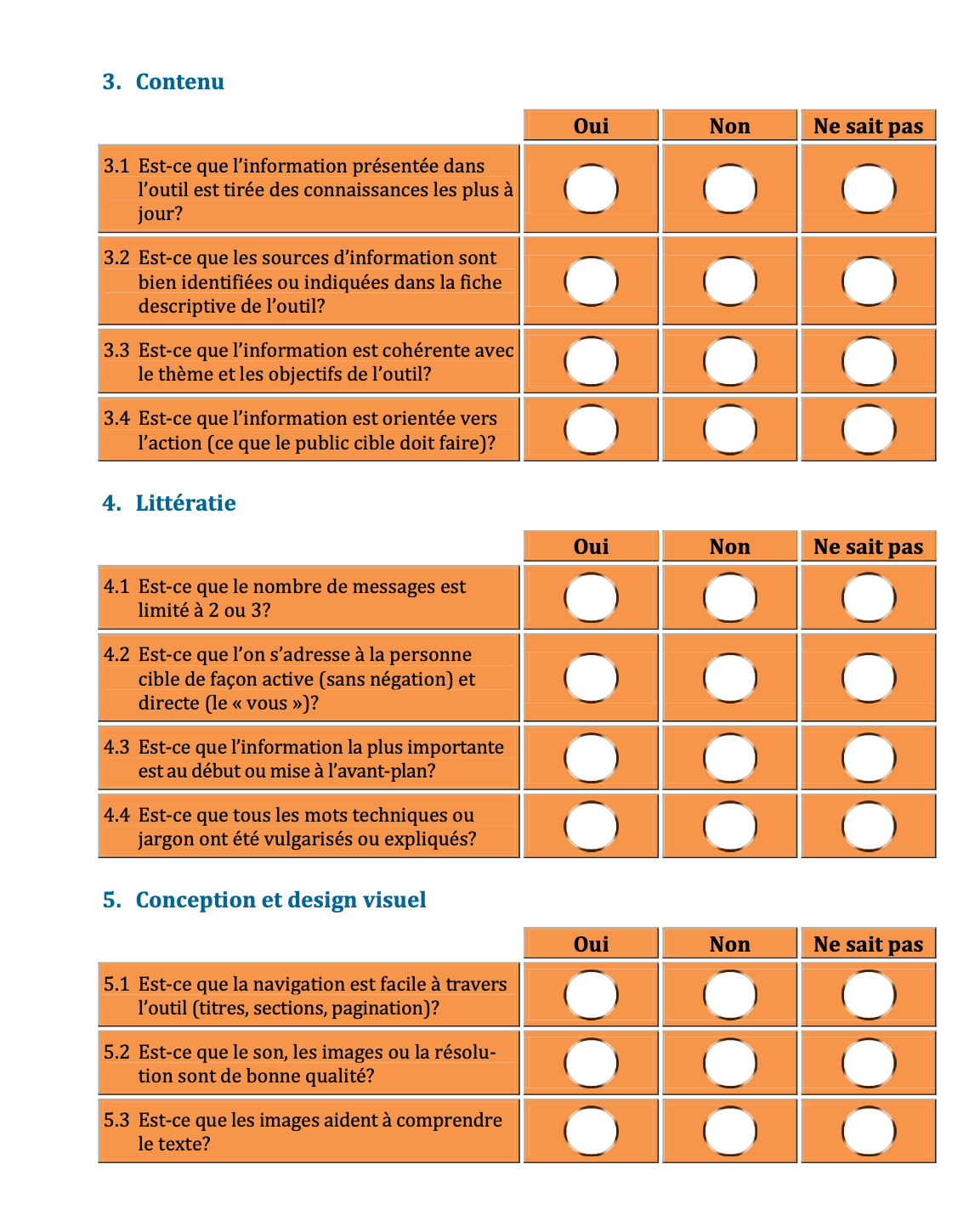
**

**
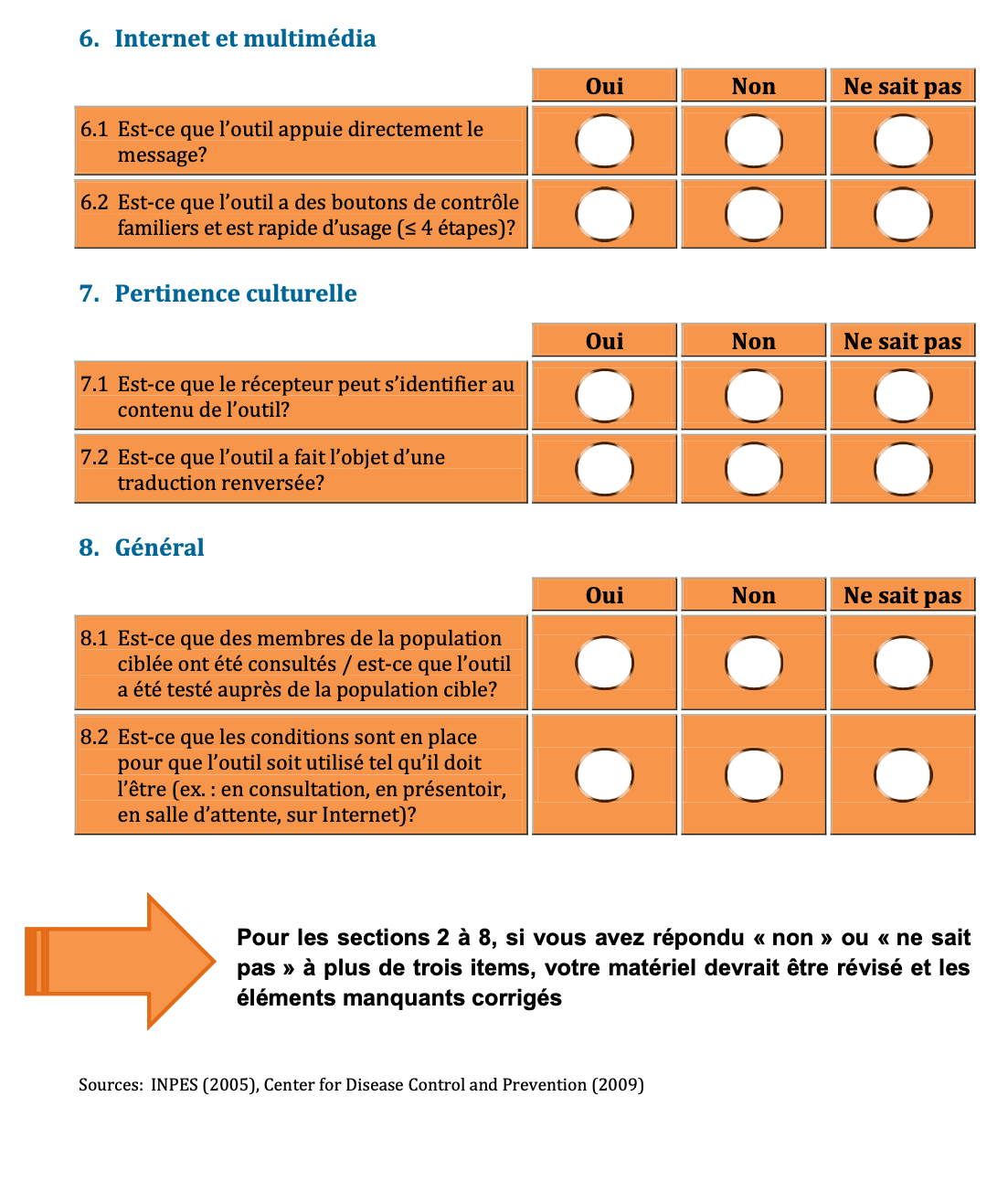
**
