# Supplementary material for: Design of a bilingual (FR-UR) website on the sensitive topic of sexual and mental health with Urdu speakers in a Parisian suburb: a qualitative study
Source: BMC Public Health. 2024 Apr 17;24:1075. doi: 10.1186/s12889-024-18479-w (PMC11025278; doi:10.1186/s12889-024-18479-w)
Supplement: Supplementary file 4 — Supplementary Material 4 [file 12889_2024_18479_MOESM4_ESM.docx]

**Focus group Guide**

1. **Pre-session briefing with interpreter on content**
2. **Briefing with participants on the purpose of the focus group, how it will be run, the role of the moderator, presentation of the project, etc.**

Framework to be set by the facilitator :

- On his/her role (facilitating exchanges, interpreting, intercultural mediator) ;

- And on the objective of this focus group :

- To reflect together on content created in advance, with a concerned person, to discuss and exchange our knowledge and representations, to produce content that respects people.
- (Specify that the aim of this work is to enable access to information for each person in relation to their needs, and to enable their empowerment, their ability to act on their health (by having access to external resources)).
- Set up the room : in a circle

Groups :

- Facilitator => health professional/intercultural interpreter

- Observer : SJ

- 1 healthcare professional per focus group

- 4 to 6 people involved

- Request authorization for video recording of focus groups.
- Estimated time: 1H45

HP : Health professionals,

P : Participants

Facilitator's key role in talking about "sensitive" subjects :

- Explain, no judgments or evaluations

- Safe space for exchange,

- The facilitator must move the conversation forward if a difficulty is observed or an exchange is problematic.

**3. Debriefing with interpreter, observer/facilitator and HP**

| Questions Focus group | Intentions |
| --- | --- |
| Presentation of each part  (Reflect on how => animation technique in relation  with confidence-building)  Presentation of the Musafir project in relation to the study, the various stages completed to date | Creating a group dynamic  Building confidence: climate |
| General health question :  How do you perceive health?  Participants, then to healthcare professionals (HP)   - Present the general introduction to the site: Script by Dr Walid   What is health prevention?   - Relaunch: Are you familiar with the term screening?   (Colorectal cancer screening, HIV and STI screening) |  Knowledge about health in general: relationship with health and health care  Objective: don't talk about sensitive subjects right away => so as not to upset them.   Knowledge of the principle of prevention: acting upstream of health problems, preventing the onset of disease.  Objective: to situate the focus group, the content of which is designed to prevent illness by providing access to information in the first place. |
| Health rights :  Do you think health is a right? (Interaction between HP and P)  (As you go along, move towards sexual health promotion rights) |  |
| Sexual health promotion :  What does sexual health promotion or sexual education mean to you?  (Relaunch: in your country of origin, did you have any information on this subject?)   Video Script: Welcome to the sexual health section  Why is this a taboo subject?  How do you find out about this subject?  What would you expect from this site in terms of sex education?  Relaunch : For example, information on where to find condoms? |  Knowledge of sexual health promotion  Objective: Introduce the theme of the content we'll be discussing   Reflecting on the notion of "taboo  Objective: to initiate reflection on the taboo of sexuality  Objective: find out about their resources and experiences in accessing information on sexual health promotion.   Expectations of those concerned  Objective: find out their expectations before distributing and showing what has been created. |
| Drawer 1: Knowledge everyone should have access to  When you were young, did you have a space to discuss your sexual health?   Puberty  At this age, young people are often introduced to pornography,   Pornography  What does it appeal to?   Sexual Desire   Pleasure   Consent   Rape   Sexual violence   Social networks | Objective:  What do these scripts remind you of?  Do you feel comfortable with this ...?  Quality of content, questions to ask:  - Can you tell me again, in your own words, what was said in the document?  - Can you show me any sentences or words that you think are difficult to understand?  - If you had to remove one or more parts, what would they be?  - What did you like best about the document?  - In this document, what did you like least? What do you think?  - What could be changed to improve it?  Questions to ask at the end of each document  - Would you share this document with your family and friends? Why or why not?  - What changes would you make before showing it to your friends and family?  - How do you feel about this document? |
| Drawer 2: More specific knowledge   Anatomy   Reproduction   First time   Contraception   Pregnancy   Abortion   STIS   Screening   Condoms   Masturbation | What do these scripts remind you of?  Do you feel comfortable with this ...?  Quality of content, questions to ask:  - Can you tell me again, in your own words, what was said in the document?  - Can you point out any phrases or words that you think are difficult to understand?  - If you had to remove one or more parts, what would they be?  - What did you like best about the document?  - In this document, what did you like least? What do you think?  - What could be changed to improve it?    - Would you share this document with your family and friends? Why or why not?  - What changes would you make before showing it to your friends and family?  - How do you feel about this document? |
| Drawer 3: Useful additional knowledge   Forced marriage,   Homosexuality,   Need for privacy (roommates) | What do these scripts remind you of?  Do you feel comfortable with this ...?  **Quality of content, questions to ask:**  - Can you tell me again, in your own words, what was said in the document?  - Can you point out any phrases or words that you think are difficult to understand?  - If you had to remove one or more parts, what would they be?  - What did you like best about the document?  - In this document, what did you like least? What do you think?  - What could be changed to improve it?  - Would you share this document with your family and friends? Why or why not?  - What changes would you make before showing it to your friends and family?  - How do you feel about this document? |
| Do you have anything to add? Any comments? Any ideas? On topics of sex education that we haven't covered? | **Objective:** Bring out other elements not covered in the content |

Questions related to the QUEBEC health literacy guide for each document/script/video.

| Information comprehension | Can you tell me again, in your own words, what was said in the document? |
| --- | --- |
| Legibility of information | Can you point out any sentences or words that you think are difficult to understand? |
| Usefulness of information | If you had to remove one or more parts, what would they be? |
| General perception | What did you like best about the document?  What did you like least about this document?  What could be changed to improve it? |
| Cultural relevance | Would you share this document with your family and friends? Why or why not?  What changes would you make before showing it to your friends and family?  How do you feel about this document? |

Source : Montréal : Agence de la santé été des services sociaux de Montréal-Quebec, 2013, 61p.
